# Dermoid cyst of the urinary bladder as a differential diagnosis of bladder calculus: a case report

**DOI:** 10.1186/1752-1947-1-32

**Published:** 2007-06-26

**Authors:** Linus I Okeke, Gabriel O Ogun, Blessing R Etukakpan, Anselmn Iyama, Adewunmi O Adeoye, Babatunde M Duduyemi

**Affiliations:** 1 Urology Division, Department of Surgery, College of Medicine, University of Ibadan and University College Hospital, PMB 5116, Ibadan, Nigeria; 2 Pathology Department, College of Medicine, University of Ibadan and University College Hospital, PMB 5116, Ibadan, Nigeria

## Abstract

Dermoid cysts are extremely rare in the urinary bladder and can pose a diagnostic dilemma to both the Urologist and the Histopathologist. Only a few cases were found documented and cited in PubMed. We present a case of dermoid cyst in the urinary bladder presenting as a bladder stone with a brief review of the literature.

## Background

Dermoid cysts are benign 'tumours', which are considered as developmental anomalies. They consist of tissue from more than one germ cell layer and occur most commonly in the ovaries but may also be found at other sites, especially in the midline and para-axial locations. They are rare in the urinary bladder The parthenogenic theory, which suggests an origin from primordial germ cell, is now the most widely accepted theory of pathogenesis of dermoid cysts. We present a case of dermoid cyst in the urinary bladder of a 34-year old woman.

## Case presentation

A 34-year-old woman presented with a 9-year history of irritative lower urinary tract symptoms (LUTS) characterized by frequency, nocturia, urgency, and urge incontinence. She also had dysuria and suprapubic pain relieved by voiding. There was no haematuria, obstructive LUTS or weight loss. She had worked in a dye industry for 3 years in the Democratic Republic of the Congo. She walked with a gliding gait suggestive of an irritating bladder stone. An abdominopelvic ultrasound scan revealed that the upper urinary tract was normal, with multiple tiny echogenic structures casting acoustic shadows in the urinary bladder. At urethrocystoscopy, a single bladder calculus adherent to the midline of the anterior wall/dome of the bladder was found, with evidence of surrounding cystitis. The urethra was normal. She received antibiotics for culture-proven *E. coli *urinary tract infection preoperatively.

**Figure 1:**
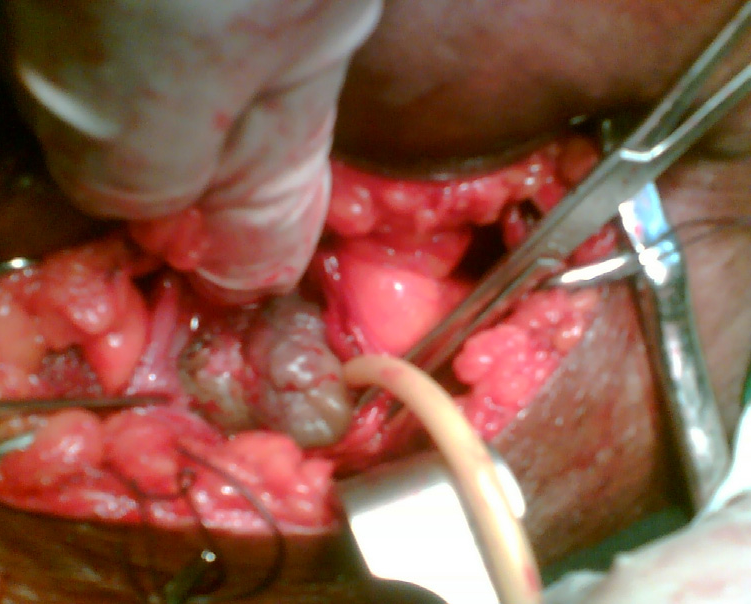
Intra operative photograph showing the "bladder mass" arising from the anterior wall of the bladder.

At an open bladder exploration 18 days later, a single grey sessile polypoid mass measuring about 5 cm diameter (Figure 1), covered with grains of whitish deposits was found arising from the midline of the anterior bladder wall. The rest of the bladder mucosa was normal. The mass was excised with a 1 cm rim of normal bladder mucosa and sent for histological examination.

**Figure 2:**
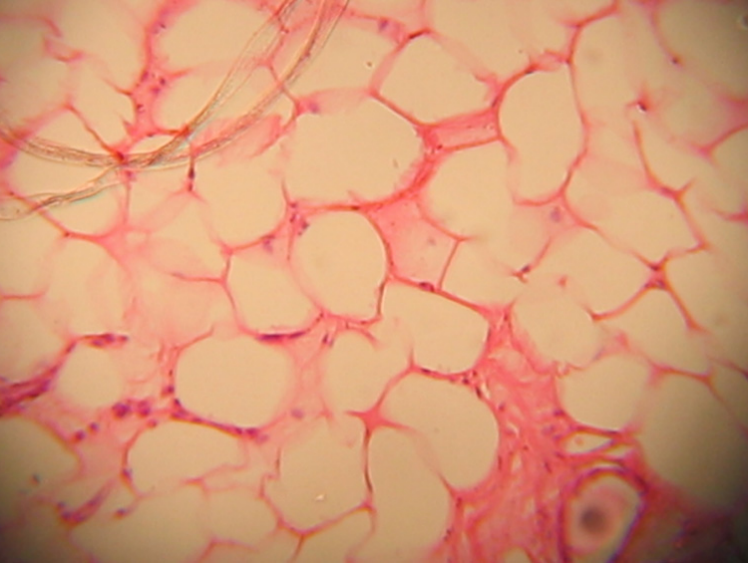
Lobules of mature adipocytes.

**Figure 3:**
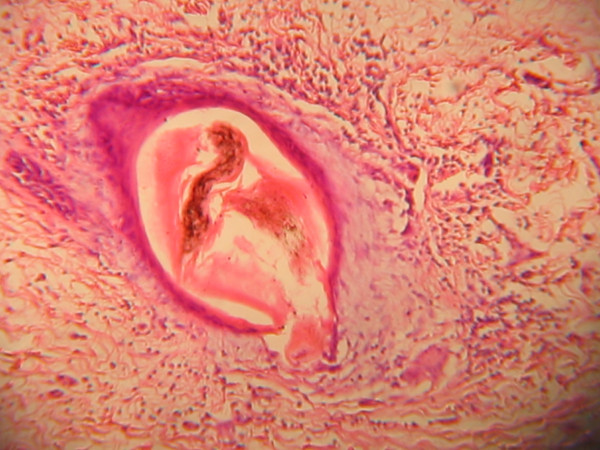
Hair follicle.

**Figure 4:**
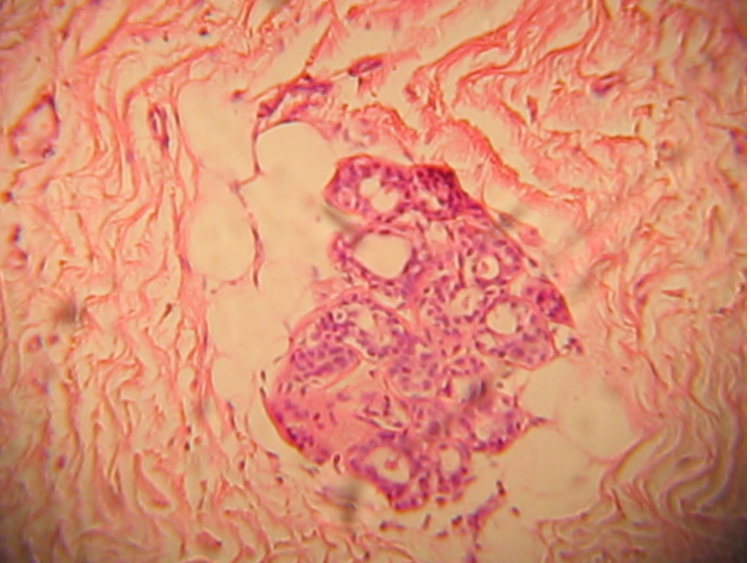
Sweat glands, hyalinized fibroblastic tissue.

**Figure 5:**
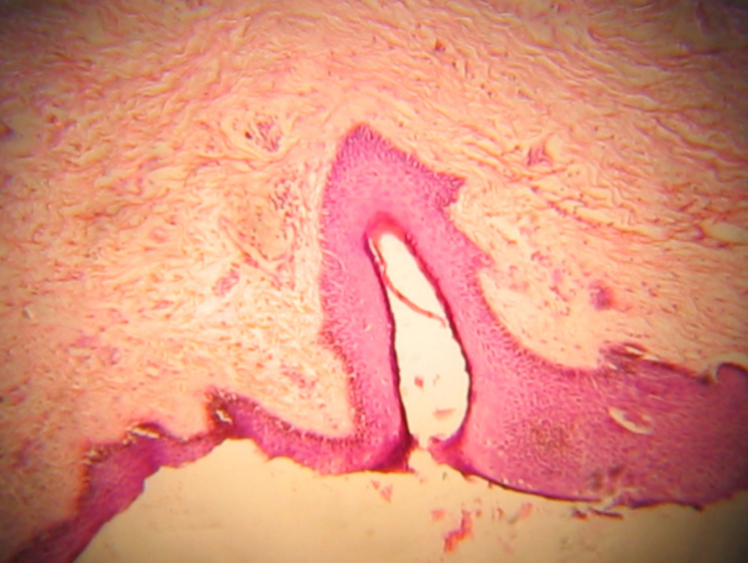
Stratified squamous epithelium, papillary and reticular dermis.

The specimen measured 4 × 2.5 × 2 cm and was greyish brown in appearance after immersion in 10% buffered formalin. It weighed 10 g. Its cut surface showed a yellowish appearance with a calculus within it. The sections (Figures 2, 3, 4, 5) showed skin tissue consisting of stratified squamous epithelium, papillary and reticular dermis, skin adnexial structures including sweat glands and hair follicles. Interspersed between were lobules of mature adipocytes, hyalinized fibroblastic tissue, blood vessels and supporting stroma.

## Discussion

Midline teratomas presumably result from abnormal germ cells when the neural tube closes at about the 3rd to 5th week of embryonic life[1,2]. A dermoid cyst in the urinary bladder is an exceedingly rare 'tumour'[3]'. We found only five cases reported and cited in the literature [4-8]. They usually contain hair and calcified material [4]. They may also be associated with bladder diverticuli and vesical stones [5]. This tumour was a solitary tumour at the apex of the bladder. It contained calcified material and fat. The anterior midline position of the bladder mass in this patient was suggestive of a dermoid cyst. Histology confirmed skin, skin adnexial structures (sweat glands, hair follicles) adipose tissue and fibroblastic tissue. The histopathological findings, which posed a diagnostic dilemma, were consistent with those of a dermoid cyst. This finding is important in that it enters the differential diagnosis of bladder mass, and the patient as well as the surgeon can be reassured since it is benign and will not need further treatment.

## Conclusion

If a "bladder stone" appears to be covered by mucosa, appears to be stuck to the anterior bladder wall and fails to roll around in the bladder at ultrasound or cystoscopy, a dermoid cyst should be considered as a differential diagnosis.

## Competing interests

The author(s) declare that they have no competing interests.

## Authors' contributions

LIO is the consultant urologist responsible for the patient's care and performed the excision of the dermoid cyst with EBR and IA who are surgical residents. GOO is the consultant pathologist and processed and read the slides with AAO and DBM who are pathology residents. LIO conceived the idea for this publication. All authors read, appraised and approved the final manuscript.
